# The prognostic value of baseline and early variations of peripheral blood inflammatory ratios and their cellular components in patients with metastatic renal cell carcinoma treated with nivolumab: The Δ-Meet-URO analysis

**DOI:** 10.3389/fonc.2022.955501

**Published:** 2022-09-23

**Authors:** Sara Elena Rebuzzi, Alessio Signori, Marco Stellato, Daniele Santini, Marco Maruzzo, Ugo De Giorgi, Paolo Pedrazzoli, Luca Galli, Paolo Andrea Zucali, Emanuela Fantinel, Claudia Carella, Giuseppe Procopio, Michele Milella, Francesco Boccardo, Lucia Fratino, Roberto Sabbatini, Riccardo Ricotta, Stefano Panni, Francesco Massari, Mariella Sorarù, Matteo Santoni, Alessio Cortellini, Veronica Prati, Hector Josè Soto Parra, Francesco Atzori, Marilena Di Napoli, Orazio Caffo, Marco Messina, Franco Morelli, Giuseppe Prati, Franco Nolè, Francesca Vignani, Alessia Cavo, Giandomenico Roviello, Miguel Angel Llaja Obispo, Camillo Porta, Sebastiano Buti, Giuseppe Fornarini, Giuseppe Luigi Banna

**Affiliations:** ^1^ Medical Oncology Unit, Ospedale San Paolo, Savona, Italy; ^2^ Department of Internal Medicine and Medical Specialties (Di.M.I.), University of Genova, Genova, Italy; ^3^ Department of Health Sciences, Section of Biostatistics, University of Genova, Genova, Italy; ^4^ SS Oncologia Medica Genitourinaria, Fondazione IRCCS Istituto Nazionale dei Tumori, Milano, Italy; ^5^ Department of Medical Oncology, Università Campus Bio-Medico of Roma, Rome, Italy; ^6^ Oncology Unit 1, Istituto Oncologico Veneto IOV - IRCCS, Padova, Italy; ^7^ Department of Medical Oncology, IRCCS Istituto Romagnolo per lo Studio dei Tumori (IRST) “Dino Amadori”, Meldola, Italy; ^8^ Department of Internal Medicine and Medical Therapy, University of Pavia, Pavia, Italy; ^9^ Medical Oncology Unit, IRCCS Policlinico San Matteo, Pavia, Italy; ^10^ Medical Oncology Unit 2, Azienda Ospedaliera Universitaria Pisana, Pisa, Italy; ^11^ Department of Biomedical Sciences, Humanitas University, Milano, Italy; ^12^ Department of Oncology, IRCCS, Humanitas Clinical and Research Center, Milano, Italy; ^13^ Department of Oncology, Azienda Ospedaliera Universitaria Integrata di Verona, University of Verona, Verona, Italy; ^14^ Division of Medical Oncology, IRCCS Istituto Tumori “Giovanni Paolo II”, Bari, Italy; ^15^ Academic Unit of Medical Oncology, IRCCS Ospedale Policlinico San Martino of Genova, Genova, Italy; ^16^ Department of Medical Oncology, Centro di Riferimento Oncologico di Aviano CRO-IRCCS, Aviano, Italy; ^17^ Medical Oncology Unit, Department of Oncology and Hemathology, University Hospital of Modena, Modena, Italy; ^18^ Oncology Unit, IRCCS MultiMedica, Milan, Italy; ^19^ Medical Oncology Unit, ASSTl– Istituti Ospitalieri Cremona Hospital, Cremona, Italy; ^20^ Medical Oncology, IRCCS Azienda Ospedaliero-Universitaria di Bologna, Bologna, Italy; ^21^ Department of Experimental, Diagnostic and Specialty Medicine, S. Orsola-Malpighi University Hospital, University of Bologna, Bologna, Italy; ^22^ U. O. Oncologia, Ospedale di Camposampiero, Padova, Italy; ^23^ Oncology Unit, Macerata Hospital, Macerata, Italy; ^24^ Department of Surgery and Cancer, Imperial College London, Faculty of Medicine, Hammersmith Hospital, London, United Kingdom; ^25^ Department of Biotechnology and Applied Clinical Sciences, University of L’Aquila, L’Aquila, Italy; ^26^ Department of Medical Oncology, Ospedale Michele e Pietro Ferrero, Verduno, Italy; ^27^ Department of Oncology, Medical Oncology, University Hospital Policlinico-San Marco, Catania, Italy; ^28^ Medical Oncology Department, University Hospital, University of Cagliari, Cagliari, Italy; ^29^ Department of Urology and Gynecology, Istituto Nazionale Tumori IRCCS Fondazione G. Pascale, Napoli, Italy; ^30^ Medical Oncology Department, Santa Chiara Hospital, Trento, Italy; ^31^ UOC Oncologia Medica, Istituto Fondazione G. Giglio, Cefalù, Italy; ^32^ Oncology Department, Gemelli Molise, Campobasso, Italy; ^33^ Department of Oncology and Advanced Technologies AUSL - IRCCS, Reggio Emilia, Italy; ^34^ Medical Oncology Division of Urogenital and Head and Neck Tumors, IEO, European Institute of Oncology IRCCS, Milano, Italy; ^35^ Division of Medical Oncology, Ordine Mauriziano Hospital, Torino, Italy; ^36^ Oncology Unit, Villa Scassi Hospital, Genova, Italy; ^37^ Department of Health Sciences, Section of Clinical Pharmacology and Oncology, University of Firenze, Firenze, Italy; ^38^ Medical Oncology Unit 1, IRCCS Ospedale Policlinico San Martino, Genova, Italy; ^39^ Interdisciplinary Department of Medicine, University of Bari “A. Moro”, Bari, Italy; ^40^ Division of Medical Oncology, A.O.U. Consorziale Policlinico di Bari, Bari, Italy; ^41^ Medical Oncology Unit, University Hospital of Parma, Parma, Italy; ^42^ Department of Medicine and Surgery, University of Parma, Parma, Italy; ^43^ Department of Oncology, Portsmouth Hospitals University NHS Trust, Portsmouth, United Kingdom

**Keywords:** renal cell carcinoma, immunotherapy, dynamics, inflammatory, NLR, prognostic

## Abstract

**Background:**

Treatment choice for metastatic renal cell carcinoma (mRCC) patients is still based on baseline clinical and laboratory factors.

**Methods:**

By a pre-specified analysis of the Meet-URO 15 multicentric retrospective study enrolling 571 pretreated mRCC patients receiving nivolumab, baseline and early dynamic variations (Δ) of neutrophil, lymphocyte, and platelet absolute cell counts (ACC) and their inflammatory ratios (IR) were evaluated alongside their association with the best disease response and overall (OS) and progression-free survival (PFS). Multivariable analyses on OS and PFS between baseline and Δ ACC and IR values were investigated with receiving operating curves-based cut-offs.

**Results:**

The analysis included 422 mRCC patients. Neutrophil-to-lymphocyte ratio (NLR) increased over time due to consistent neutrophil increase (p < 0.001). Higher baseline platelets (p = 0.044) and lower lymphocytes (p = 0.018), increasing neutrophil Δ (p for time-group interaction <0.001), higher baseline IR values (NLR: p = 0.012, SII: p = 0.003, PLR: p = 0.003), increasing NLR and systemic immune-inflammatory index (SII) (i.e., NLR x platelets) Δ (p for interaction time-group = 0.0053 and 0.0435, respectively) were associated with disease progression. OS and PFS were significantly shorter in patients with baseline lower lymphocytes (p < 0.001 for both) and higher platelets (p = 0.004 and p < 0.001, respectively) alongside early neutrophils Δ (p = 0.046 and p = 0.033, respectively). Early neutrophils and NLR Δ were independent prognostic factors for both OS (p = 0.014 and p = 0.011, respectively) and PFS (p = 0.023 and p = 0.001, respectively), alongside baseline NLR (p < 0.001 for both) and other known prognostic variables.

**Conclusions:**

Early neutrophils and NLR Δ may represent new dynamic prognostic factors with clinical utility for on-treatment decisions.

## 1 Introduction

Immune checkpoint inhibitors (ICIs) have reshaped the treatment landscape of metastatic renal cell carcinoma (mRCC) with the introduction of nivolumab in pretreated patients in 2015 and the more recent first-line immunotherapy-based combinations (1–3).

Despite the survival benefit leading to these new immunotherapy indications, the proportion of mRCC patients achieving long-term benefits from ICI-based therapies is still low. Early predictive biomarkers are needed to optimize patient and treatment selection (4, 5). The programmed-cell-death-ligand1 (PD-L1) expression, tumor mutational burden (TMB), and tumor microenvironment-related signatures have been investigated for their prognostic and predictive value. However, none has still reached sufficient evidence or applicability to be routinely tested in everyday clinical practice (6–9). Although PD-L1 expression correlated with poor prognosis and advanced clinicopathological features in RCC patients (10–12), it is expressed in about one quarter of patients with clear-cell RCC and approximately 10% of those with non-clear cell RCC (10) and does not seem to have a predictive value (13).

Inflammatory ratios (IR) from peripheral blood might reflect the cancer-related inflammatory phenomena, the host immune response to cancer and comorbidity (14). In practically every area of medicine, including cancer patients, elements of the full blood count, like the total leukocyte, neutrophil, lymphocyte, monocyte, and platelet counts, have been extensively studied as a proxy of a dysfunctional pro-inflammatory response (15–17). It has long been known that blood count parameters have a prognostic value for mRCC. High neutrophils were initially reported as a poor prognostic indicator in 1996 (18). Later, the notion of neutrophils-to-lymphocytes ratio (NLR) reached the clinical practice (19). No later than 2011, the relevance of an elevated platelet count was recognized (20). IR have emerged as a quick and inexpensive assessment with reproducible prognostic value across different tumor types, stages, and treatment settings, particularly for patients with metastatic tumors treated with ICIs (21, 22). However, their baseline value has been mainly investigated so far, while increasing evidence suggests a possible correlation with disease outcome related to their early variations during treatment, particularly in lung cancer patients (23–33). If associated with worse prognosis and failure of therapy their early variations might have clinically helpful aftermaths, like the anticipation of disease reassessments during treatments and an earlier start of the next treatment line. Furthermore, a better understanding of the IR specific cellular component on-treatment variations would shed light on the shift of the patient’s immune system in response to anti-tumoral treatments, specifically the ICIs.

The Meet-URO 15 study is one of the largest analyses of baseline prognostic factors, including IR in patients with mRCC treated with ICIs (34). This study developed a novel prognostic score, namely the Meet-URO score, based on the addition of two newly identified independent variables, or the NLR and the presence of bone metastases.

In this pre-specified sub-analysis of the Meet-URO 15 study, we longitudinally investigated the dynamics of neutrophil, lymphocyte and platelet absolute cell counts (ACC) and IR during the first four nivolumab treatment administrations and their correlation with response and survival.

## 2 Materials and methods

The analysis was a pre-specified secondary analysis of the multicentric retrospective Meet-URO 15 study, approved by the institutional review board (regional ethical committee of Liguria – registration number 068/2019). The Meet-URO 15 study was conducted among 34 Italian centers and enrolled 571 mRCC patients. It was performed according to the Declaration of Helsinki. All living patients signed written informed consent.

### 2.1 Study population and treatment

Patients with mRCC who had received at least two completed nivolumab administrations as ≥2^nd^ treatment line between October 2015 and November 2019 were included in the analysis. Nivolumab was administered intravenously at the dose of 3 mg/kg every 2 weeks until May 2018, then at the fixed dose of 240 mg every 2 weeks, or 480 mg every 4 weeks, according to the clinical practice of each participating center. The treatment was continued until progressive disease (PD), unacceptable toxicity, death, or patient choice. Patients with radiological PD were allowed to continue therapy beyond progression of clinical benefit according to physicians’ decision.

The follow-up consisted of periodic physical examinations, laboratory analyses, and imaging assessments. Radiological assessments included computed tomography (CT) scan of chest-abdomen-pelvis and head (when clinically indicated) at baseline and every 2–4 months thereafter, according to physicians’ practice, or when PD was clinically suspected.

### 2.2 Absolute cell counts and inflammatory ratios from peripheral blood

Data from full blood counts performed within 7 days from each of the first four nivolumab administrations were collected, including neutrophils, lymphocytes and platelets ACC, and the following IR: neutrophil-to-lymphocyte ratio (NLR), platelet-to-lymphocyte ratio (PLR) and the systemic immune-inflammation index (SII, calculated as NLR × platelets as originally developed) (35). Patients were then followed up until the date of the database lock for the final analysis on 31 July 2020.

### 2.3 Study objectives and endpoints

The first study objective was the description of the ACC and IR value variations through the first four nivolumab administrations (Delta, Δ). The Delta was derived from subtracting the parameter value at the fourth nivolumab administration minus baseline level. The second study objective evaluated the correlation between ACC and IR baseline and Δ values with the best disease response to treatment. The third study objective included the correlation of their baseline and early Δ values with overall survival (OS) and progression-free survival (PFS), the assessment of related prognostic models and potential interactions between baseline and Δ values on OS and PFS. Early Δ was defined as the variations of values from the first to the second treatment administrations, or the subtraction of the parameter value at the second nivolumab administration minus baseline level. The disease response to treatment was defined in each center, referring to the Response Evaluation Criteria in Solid Tumours (RECIST) criteria version 1.1 as complete response (CR), partial response (PR), stable disease (SD) and PD (36). Responders were defined as those patients achieving CR or PR as the best disease response. OS was calculated from the first nivolumab administration until death, censored at last follow-up for living patients, while PFS was calculated from the first nivolumab administration until PD or death, censored at last follow-up for patients who did not progress and were alive at the end of the follow-up.

### 2.4 Statistical analysis

Patients’ characteristics were reported using absolute frequency and percentage for categorical variables and by mean with standard deviations, or median and ranges, for quantitative variables.

Analysis of variance (ANOVA) was used to test differences between baseline ACC, IR, and the best response to treatment; p values for each comparison (i.e., PD vs. CR/PR) were adjusted using the false discovery rate approach for multiple comparisons.

The longitudinal trend of ACC and IR was assessed using the linear mixed model with random intercept; p-values for longitudinal trends were corrected for multiple comparisons using the false discovery rate approach. The interaction between the therapy administration number and best response to treatment was performed to test differences across administrations between CR/PR, SD and PD, and ACC or IR.

The Kaplan–Meier method was used to estimate survival curves of OS and PFS by the baseline and early ACC and IR Δ values.

Survival receiver operating curves (ROC) based on OS were performed to identify both baseline ACC and early ACC and IR Δ cut-off values; baseline IR cut-offs were those identified in the previous analysis (34).

Univariable and multivariable analyses to test the association between baseline, early ACC, and IR Δ values and PFS and OS were performed using the Cox proportional hazard regression model. As the early Δ was the variable of interest, multivariable models were performed only for those values with a p value <0.10 at the univariable analyses. All the other characteristics, including the International Metastatic RCC Database Consortium (IMDC) risk score for mRCC and the presence of bone metastases, were also considered into the model when a p value <0.10 was found at the univariable analysis.

The interaction between baseline and early Δ values was assessed to test whether the association with outcomes depended on baseline values. The level of significance was set to 0.05.

All statistical analyses were performed using Stata v.16 (StataCorp 2019).

## 3 Results

### 3.1 Patients’ characteristics

**Figure 1:**
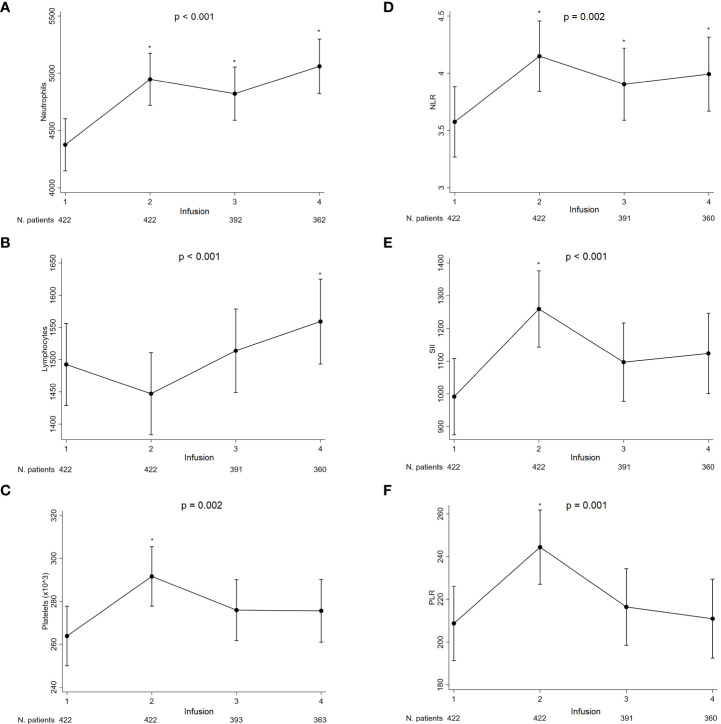
The ACC and IR Δ values through the first four nivolumab administrations.Neutrophils **(A)**, lymphocytes **(B)**, platelets **(C)**, NLR **(D)**, SII **(E)** and PLR **(F)** were assessed.*Significant difference compared with baseline and adjusted for multiple comparisons using the false discovery rate approach.

Four hundred twenty-two mRCC patients had available data for the analysis. The CONSORT flow diagram is shown in **Figure 1S**. Forty-two out of the 571 overall patients (7.4%) did not reach the second treatment cycle. Of 107 patients (18.7%) who received at least two treatment cycles, 40 and 67 had laboratory missing data at baseline or the second cycle, respectively, thus leading to the 422 patients included in the analysis. Their characteristics are reported in **Table 1**. Of the 422 patients, 309 (73.2%), 82 (19.4%), and 31 (7.4%) received nivolumab as a second-, third-, or further line treatment. Most patients had clear-cell histology (85%) and received nivolumab as a second line treatment (73%); median age was 63.4 years (range: 18–85). According to the prognosis estimation at metastatic disease onset, 34% of patients were at favorable, 60% intermediate, and 6.5% poor-risk by the IMDC classification, while 22% belonged to the Meet-URO score risk group 1, 43% to group 2, 23% to group 3, and 11% to group 4.

**Table 1:** Patients’ characteristics.

Patients n = 422	
*Characteristics*	*N (%)*
Gender	
Male	305 (72.3)
Female	117 (27.7)
Median age, years (range)	63.4 (18-85)
<70	314 (74.4)
≥70	108 (25.6)
Karnofsky performance status	
‗80%	367 (87.0)
<80%	55 (13.0)
Histologic subtype	
Clear cell	358 (84.8)
Non-clear cell	64 (15.2)
Nephrectomy	
Yes No	376 (89.1)46 (10.9)
Metastatic ad diagnosis	
Yes	174 (41.2)
No	248 (58.8)
IMDC score at metastatic diagnosis	
Favorable	130 (33.9)
Intermediate	229 (59.6)
Poor	25 (6.5)
Missing	38
Meet-URO score	
1 (0-1)	92 (21.9)
2 (2-3)	182 (43.3)
3 (4-5)	98 (23.4)
4 (6-8)	48 (11.4)
5 (9)	0
Nivolumab line	
2^nd^ line	309 (73.2)
3^rd^ line	82 (19.4)
≥ 4^th^ line	31 (7.4)
IMDC score at start of nivolumab	
Favorable	92 (21.9)
Intermediate	280 (66.7)
Poor	48 (11.4)
Missing	2
Lymph-nodal metastases	
Yes	226 (53.6)
No	196 (46.5)
Visceral metastases	
Yes	385 (91.2)
No	37 (8.8)
Bone metastases	
Yes	147 (34.8)
No	275 (65.2)

N, number of patients; IMDC, International Metastatic RCC Database Consortium.

### 3.2 Absolute cell count and inflammatory ratio variations during treatment

The ACC and IR Δ values through the first four nivolumab administrations are represented in **Figure 1**. Among the formers, the neutrophil counts consistently increased from baseline (mean: 4313 x10e3/L) to the fourth administration (mean: 5058x10e3/L) with a significant positive Δ at each therapy administration (p < 0.001; **Figure 1A**).

After a non-significant initial drop below the baseline value (mean: 1492x10e3/L), lymphocyte counts progressively increased with a significant positive Δ reached at the fourth administration (mean: 1559x10e3/L) (p = 0.030) (**Figure 1B**).

A significant platelet positive Δ from baseline count (mean: 264x10e9/L) was observed at the second administration (mean: 292x10e9/L) (p = 0.003), followed by a non-significant drop with counts remaining higher than baseline until the fourth administration (mean: 276x10e9/L) (**Figure 1C**).

Reflecting trends of their constituting cell types, a significantly positive Δ was observed at each therapy administration time point for the NLR (from baseline mean 3.58 to 3.99 at the fourth; p < 0.001, p = 0.037, p = 0.015 at the second, third, and fourth, respectively) (**Figure 1D**), at the second only for SII (from mean 992 to 1260; p < 0.001) (**Figure 1E**) and PLR (from mean 209 to 244 at the second; p = 0.001) (**Figure 1F**).

### 3.3 Absolute cell counts and inflammatory ratios according to disease response

#### 3.3.1 Baseline values

**Figure 2:**
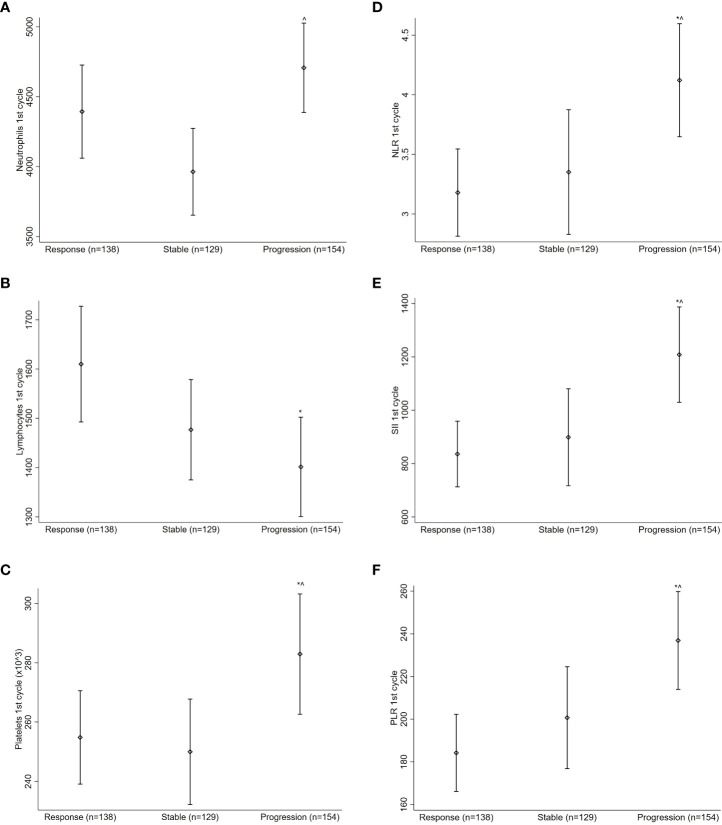
The baseline ACC and IR values according to the disease response to nivolumab.Neutrophils **(A)**, lymphocytes **(B)**, platelets **(C)**, NLR **(D)**, SII **(E)** and PLR **(F)** were assessed.*Significant differences compared with response (R); ^Significant difference compared with stable disease (S); 2A: p = 0.11 for S vs. R; p = 0.17 for progression (P) vs. R; p = 0.003 for P vs. S; 2B: p = 0.14 for S vs. R; p = 0.018 for P vs. R; p = 0.33 for P vs. S; 2C: p = 0.72 for S vs. R; p = 0.044 for P vs. R; p = 0.036 for P vs. S; 2D: p = 0.61 for S vs. R; p = 0.012 for P vs. R; p = 0.029 for P vs. S; 2E: p = 0.60 for S vs. R; p = 0.003 for P vs. R; p = 0.014 for P vs. S; 2F: p = 0.31 for S vs. R; p = 0.003 for P vs. R; p = 0.032 for P vs. S; p values were adjusted for multiple comparisons using the false discovery rate approach.

The baseline ACC and IR values according to the disease response to nivolumab are reported in **Figure 2**. Patients with PD had higher platelet (mean: 283x10e9/L) and lower lymphocyte (mean: 1401x10e3/L) baseline counts than responders (mean: 255 x10e9/L and 1610x10e3/L; p = 0.044 and p = 0.018, respectively) and higher baseline neutrophils (mean: 4707x10e3/L) and platelets (mean: 283x10e9/L) compared to patients with SD (mean: 3963x10e3/L and 250x10e9/L; p = 0.003 and p = 0.036, respectively) (**Figures 2A–C**).

Higher baseline NLR (mean: 4.12), SII (mean: 1208), and PLR (mean: 237) values were consistently associated with PD than responders (mean: 3.18, 836 and 184; p = 0.012, p = 0.003 and p = 0.003, respectively) or SD (mean: 3.35, 899 and 201; p = 0.029, p = 0.014 and p = 0.032, respectively) (**Figures 2D–F**).

#### 3.3.2 Longitudinal variations (Δ)

**Figure 3:**
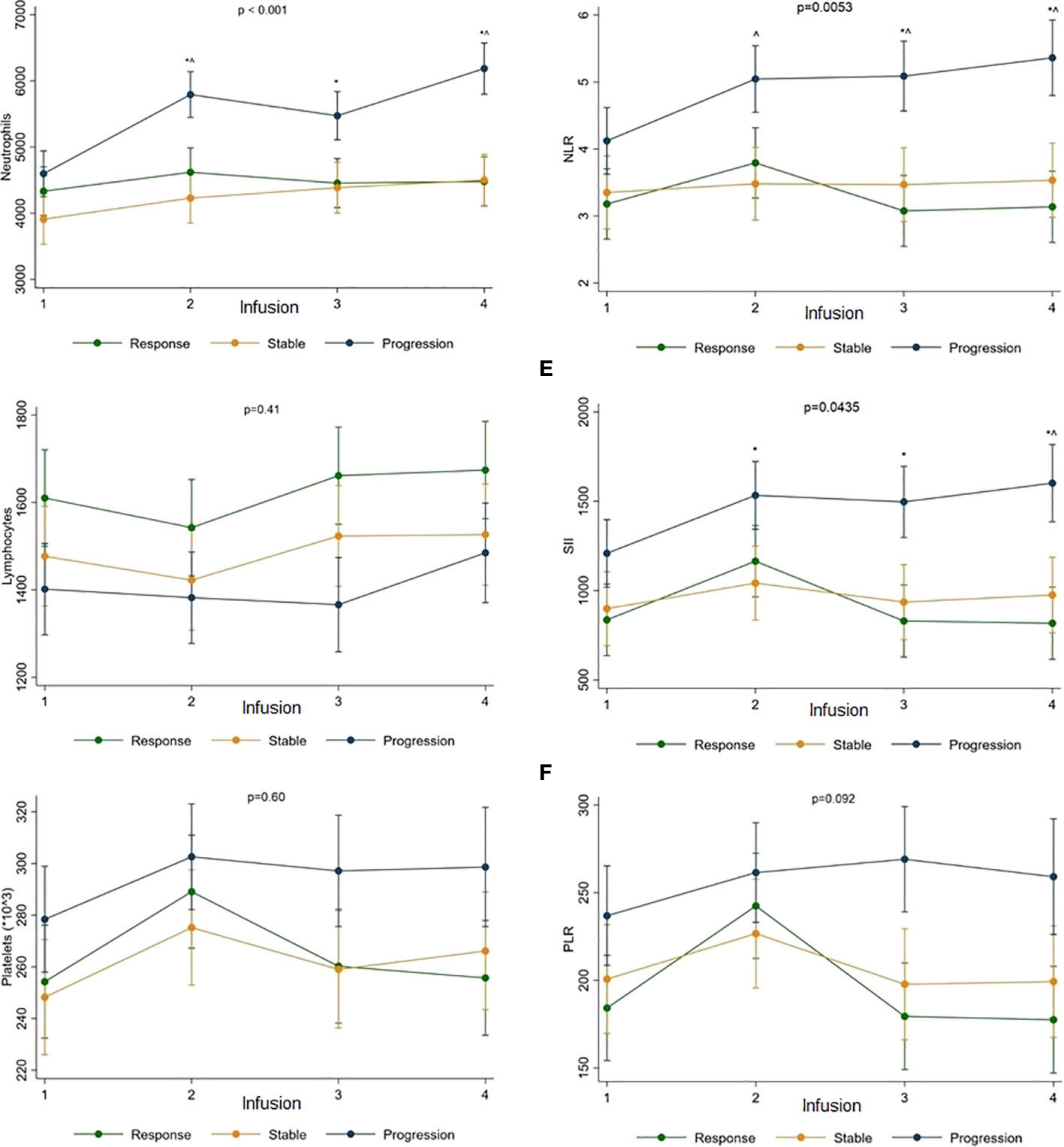
The ACC and IR value Δ according to the disease response to therapy.Neutrophils **(A)**, lymphocytes **(B)**, platelets **(C)**, NLR **(D)**, SII **(E)** and PLR **(F)** were assessed.*Significant differences compared with response; ^ Significant difference compared with stable disease.

The ACC and IR values Δ according to the disease response to therapy are represented in **Figure 3**.

Neutrophils significantly increased in patients with PD (from baseline mean count of 4612x10e3/L to 6176x10e3/L at the fourth administration) compared to responders (from 4364x10e3/L to 4547x10e3/L) or patients with SD (from 3890x10e3/L to 4498x10e3/L) (p for time-group interaction < 0.001) (**Figure 3A**).

No significant differences in lymphocyte and platelet Δ were observed according to disease response (p = 0.41 and p = 0.60, respectively). However, the higher baseline counts of lymphocytes were maintained over treatment in responders (from baseline mean count of 1591x10e3/L to 1653x10e3/L at the fourth administration) compared to patients with PD (from 1435x10e3/L to 1502x10e3/L) or SD (from 1476x10e3/L to 1525x10e3/L) (**Figure 3B**). Similarly, the higher baseline platelet counts were maintained in patients with PD (from 277x10e9/L to 298x10e9/L at the fourth administration) than responders (from 251x10e9/L to 255x10e9/L) or patients with SD (from 246x10e9/L to 266x10e9/L) (**Figure 3C**).

Accordingly, NLR and SII values significantly increased in patients with PD (from baseline mean value of 4.24 to 5.41 at the fourth administration for NLR, and from 1208 to 1618 for the SII) compared to responders (from 3.32 to 3.24 for NLR, and from 845 to 830 for SII) or patients with SD (from 3.35 to 3.55 for NLR, and from 883 to 973 for SII) (p for interaction time-group = 0.0053 and 0.0435 for NLR and SII, respectively) (**Figures 3D, E**). The PLR value Δ was not significantly increased according to the disease response (p for interaction time-group = 0.092) (**Figure 3F**).

### 3.4 Correlation of absolute cell counts and inflammatory ratios with survival outcomes

**Figure 4:**
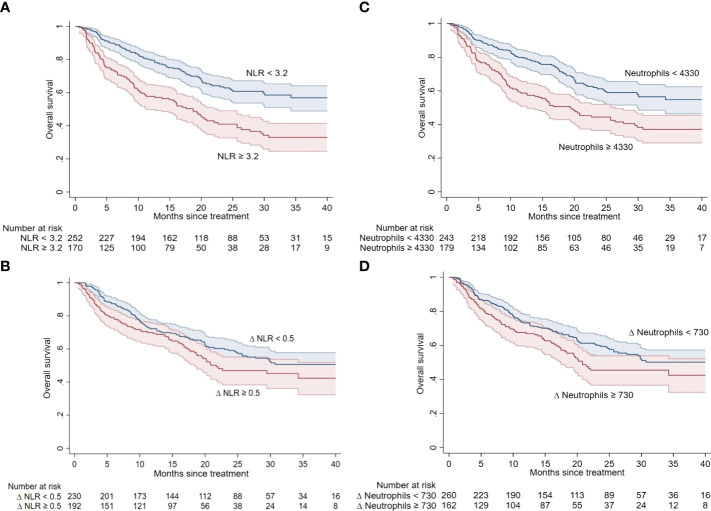
The univariable analyses of baseline and early Δ of NLR **(A, B)** and neutrophils **(C, D)**.

The univariable analyses of baseline and early ACC and IR Δ values, based on their ROC-based cut-off values, are reported in **Table 2** and represented in **Figure 4**, **2S** and **3S**.

**Table 2:** Univariable analysis on survival outcomes of absolute cell counts and immune-inflammatory indices baseline and early Δ, and baseline clinical parameters.

Inflammatory indices	ROC-based cut-off values	PFS		OS	
		mPFS(95% CI)	Univariable(HR; 95% CI; p value)	mOS(95% CI)	Univariable(HR; 95% CI; *p* value
***Absolute cell counts***					
Baseline Neutrophils (x10e3/L)	≥ 4330	6.9 (5.1-10.9)	1.25; 0.99-1.56; p = 0.059	19.4 (12.6-26.4)	1.87; 1.40-2.49; **p < 0.001**
	< 4330	10.2 (8.4-14.3)	1.00 (ref)	NR	1.00 (ref)
Early Δ Neutrophils	≥ 730	6.1 (4.7-9.2)	1.29; 1.02-1.62; **p** = **0.033**	20.8 (17.4-43.9)	1.34; 1.01-1.80; **p** = **0.046**
	< 730	11.0 (9.3-13.9)	1.00 (ref)	46.9 (25.7-NR)	1.00 (ref)
Baseline Lymphocytes (x10e3/L)	< 1460	6.4 (5.0-8.4)	1.57; 1.25-1.98; **p < 0.001**	20.0 (17.1-27.7)	1.88; 1.39-2.53; **p < 0.001**
	≥ 1460	13.9 (9.9-18.5)	1.00 (ref)	NR	1.00 (ref)
Early Δ Lymphocytes	≥ -10	8.4 (5.5-12.1)	1.10; 0.88-1.38; p = 0.41	25.7 (20.1-43.9)	1.15; 0.86-1.54; p = 0.34
	< -10	9.9 (8.1-12.5)	1.00 (ref)	46.9 (23.0-NR)	1.00 (ref)
Baseline Platelets (x10e9/L)	≥ 263	8.4 (5.1-10.1)	1.40; 1.11-1.76; **p** = **0.004**	19.4 (13.8-25.7)	1.92; 1.44-2.56; **p < 0.001**
	< 263	10.9 (7.8-15.0)	1.00 (ref)	NR	1.00 (ref)
Early Δ Platelets	≥ 17	8.5 (5.5-10.5)	1.07; 0.85-1.34; p = 0.56	26.4 (20.2-NR)	0.97; 0.73-1.30; p = 0.86
	< 17	10.8 (8.0-14.3)	1.00 (ref)	34.3 (20.8-NR)	1.00 (ref)
***Indices***					
Baseline NLR	≥ 3.2	5.8 (4.6-8.3)	1.58; 1.26-1.99; **p < 0.001**	18.7 (11.3-22.7)	2.10; 1.57-2.80; **p < 0.001**
	< 3.2	11.2 (9.5-16.6)	1.00 (ref)	NR	1.00 (ref)
Early Δ NLR	≥ 0.5	6.4 (5.0-9.3)	1.37; 1.09-1.72; **p** = **0.007**	21.7 (18.4-43.9)	1.32; 0.99-1.76; p = 0.062
	< 0.5	12.1 (9.5-16.8)	1.00 (ref)	46.9 (25.7-NR)	1.00 (ref)
Baseline SII	≥ 720	6.1 (4.7-9.4)	1.51; 1.21-1.90; **p < 0.001**	18.7 (13.8-22.0)	2.27; 1.69-3.04; **p < 0.001**
	< 720	11.3 (9.5-18.3)	1.00 (ref)	NR	1.00 (ref)
Early Δ SII	≥ 218	6.4 (4.6-9.5)	1.24; 0.99-1.57; p = 0.061	24.5 (18.7-NR)	1.22; 0.91-1.64; p = 0.18
	< 218	11.0 (9.2-14.7)	1.00 (ref)	30.7 (23.7-NR)	1.00 (ref)
Baseline PLR	≥ 176	6.5 (4.7-9.5)	1.52; 1.21-1.91; **p < 0.001**	19.9 (15.5-22.7)	2.23; 1.66-3.01; **p < 0.001**
	< 176	11.5 (9.3-16.8)	1.00 (ref)	NR	1.00 (ref)
Early Δ PLR	≥ 21	9.2 (5.9-11.3)	1.07; 0.85-1.35; p = 0.54	27.7 (19.4-NR)	1.09; 0.82-1.45; p = 0.57
	< 21	9.9 (6.9-14.3)	1.00 (ref)	30.1 (21.7-NR)	1.00 (ref)
**Baseline clinical parameter**					
Heng score	Favorable	22.5 (16.4-35.2)	1.00 (ref)	NR	1.00 (ref)
	Intermediate	8.2 (5.9-9.5)	1.85; 1.36-2.51; **p < 0.001**	25.7 (20.1-34.3)	2.83; 1.79-4.50; **p < 0.001**
	Poor	2.9 (2.2-5.5)	3.28; 2.16-4.99; **p < 0.001**	8.1 (3.7-10.7)	7.13; 4.12-12.37; **p < 0.001**
Metastatic at diagnosis	Yes	6.4 (5.3-9.3)	1.21; 0.96-1.53; p = 0.11	21.7 (17.5-34.3)	1.40; 1.05-1.87; **p** = **0.023**
	No	11.2 (9.3-14.7)	1.00 (ref)	46.9 (24.8-NR)	1.00 (ref)
Nephrectomy	Yes	9.9 (8.3-12.5)	0.60; 0.42-0.85; **p** = **0.004**	43.9 (25.7-NR)	0.43; 0.29-0.62; **p < 0.001**
	No	4.0 (2.9-8.8)	1.00 (ref)	14.5 (8.6-19.4)	1.00 (ref)
Histologic subtype	Clear-cell	9.5 (7.9-11.5)	0.95; 0.69-1.31; p = 0.77	29.5 (22.0-NR)	1.08; 0.71-1.63; p = 0.72
	Non-clear cell	6.6 (5.0-13.6)	1.00 (ref)	NR	1.00 (ref)
Lymph node metastases	Yes	7.4 (5.6-10.1)	1.15; 0.92-1.45; p = 0.22	25.7 (19.9-30.7)	1.28; 0.95-1.71; p = 0.10
	No	11.0 (8.8-13.8)	1.00 (ref)	46.9 (22.7-NR)	1.00 (ref)
Viscera metastases	Yes	9.3 (6.9-11.1)	1.09; 0.72-1.64; p = 0.69	29.8 (22.0-NR)	1.04; 0.62-1.74; p = 0.88
	No	11.3 (5.8-23.4)	1.00 (ref)	25.7 (16.7-NR)	1.00 (ref)
Bone metastases	Yes	6.4 (4.6-8.4)	1.51; 1.20-1.91; **p** = **0.001**	18.7 (13.1-25.0)	1.81; 1.36-2.42; **p < 0.001**
	No	11.3 (9.3-16.0)	1.00 (ref)	46.9 (29.8-NR)	1.00 (ref)
Line of therapy	2	9.5 (6.6-12.1)	1.00 (ref)	30.1 (21.4-NR)	1.00 (ref)
	3	9.5 (6.1-13.1)	1.06; 0.84-1.35; p = 0.61	NR	0.97; 0.71-1.31; p = 0.83
	>4	8.3 (3.2-16.6)	0.94; 0.68-1.28; p = 0.68	18.1 (9.3-NR)	0.86; 0.57-1.30; p = 0.48

Early Δ value variations between second and first therapy infusion, mOS median overall survival, mPFS median progression-free survival, NLR neutrophils-to-lymphocytes ratio, NR not reached, PLR platelets-to-lymphocytes ratio, ROC receiving operating curve, SII systemic immune-inflammatory index.

In bold, significant p-values.

#### 3.4.1 Baseline values

Higher baseline platelet (cut-off: ≥263x10e9/L) and lower lymphocyte (cut-off: <1460x10e3/L) counts were either significantly associated with worse OS (p < 0.001 for both) and PFS (p = 0.004 and p < 0.001, respectively), while higher neutrophils (≥4330x10e3/L) were significantly associated with OS (p < 0.001) only and not with PFS (p = 0.059) (**Table 2**, **Figure 1S and 2S**).

Higher NLR (cut-off: ≥3.2), SII (cut-off: ≥720), and PLR (cut-off: ≥176) baseline values were associated with both worse OS (p < 0.001 for all) and PFS (p < 0.001 for all) (**Table 2**).

#### 3.4.2 Longitudinal variations (Δ)

Increased neutrophil early Δ (cut-off: ≥730x10e3/L) only was either associated with OS (p = 0.046) or PFS (p = 0.033), while increased NLR early Δ (cut-off: ≥0.5) was significantly associated with PFS (p = 0.007) but not with OS (p = 0.062) (**Table 2**, **Figure 4**, **Figure 5S** and **Figure 6S**).

#### 3.4.3 Multivariable analysis on survival outcomes

In two prognostic models by the NLR or neutrophil counts, higher baseline NLR values (cut-off: ≥ 3.2) (p < 0.001) or neutrophils (cut-off: ≥4330x10e3/L) (p < 0.001), increased early D of NLR (cut-off: ≥0.5) (p = 0.014) or neutrophils (cut-off: ≥730x10e3/L) (p = 0.011), alongside IMDC intermediate (p < 0.001 with both models) and poor risk (p < 0.001 with both models) and the presence of bone metastases (p = 0.006 and p = 0.004, respectively) resulted as negative independent factors on OS at the multivariable analysis (**Table 3**).

**Table 3:** Multivariable analysis on OS of absolute cell counts and immune-inflammatory indices baseline and early Δ, and baseline clinical parameters.

Inflammatory indices	ROC-based cut-off values	Multivariable Cox regression for OS	
		NLR	Neutrophils
		(HR; 95% CI; *p* value)	(HR; 95% CI; p value)
Baseline NLR	≥ 3.2	1.83; 1.35-2.49; **p < 0.001**	
	< 3.2	1.00 (ref)	
Early Δ NLR	≥ 0.5	1.46; 1.08-1.96; **p = 0.014**	
	< 0.5	1.00 (ref)	
		*p* value for interaction baseline NLR and ΔNLR = 0.73	
Baseline Neutrophils	≥ 4330 x10e3/L		1.82; 1.35-2.45; **p < 0.001**
	< 4330 x10e3/L		1.00 (ref)
Early Δ Neutrophils	≥ 730 x10e3/L		1.48; 1.09-1.99; **p = 0.011**
	< 730 x10e3/L		1.00 (ref)
			*p* value for interaction baseline Neutrophils and ΔNeutrophils = 0.074
**Clinical parameter**			
IMDC score	Favorable	1.00 (ref)	1.00 (ref)
	Intermediate	2.79; 1.73-4.50; **p < 0.001**	2.68; 1.66-4.32; **p < 0.001**
	Poor	5.46; 3.03-9.82; **p < 0.001**	5.52; 3.07-9.93; **p < 0.001**
Metastatic at diagnosis	Yes	0.85; 0.61-1.18; p = 0.32	0.84; 0.60-1.17; p = 0.30
	No	1.00 (ref)	1.00 (ref)
Nephrectomy	Yes	0.67; 0.43-1.04; p = 0.077	0.57; 0.37-0.87; **p = 0.009**
	No	1.00 (ref)	1.00 (ref)
Bone	Yes	1.52; 1.13-2.04; **p = 0.006**	1.55; 1.15-2.08; **p = 0.004**
	No	1.00 (ref)	1.00 (ref)

CI confidence interval, early Δ value variations between 2^nd^ and 1^st^ therapy infusion, HR hazard ratio, IMDC International Metastatic RCC Database Consortium Risk Score for RCC, NLR neutrophils-to-lymphocytes ratio, OS overall survival, RCC renal cell carcinoma, ROC receiving operating curve.

In bold, significant p-values.

Multivariable analysis results on PFS are reported in **Table 1S** and confirmed higher baseline NLR (p < 0.001) and SII (p = 0.038) values and increased early Δ of NLR (p = 0.001) and neutrophils (p = 0.023), alongside the IMDC intermediate- and poor-risk and the presence of bone metastases as negative prognostic factors (**Table 1S**).

The Harrel’s c-index of the model with neutrophil early D was 0.692 for the OS and 0.630 for PFS, while with NLR early Δ was 0.693 and 0.644, respectively.

#### 3.4.4 Interaction on survival outcomes between absolute cell counts and early Δ

A significant interaction between increased neutrophil early Δ (cut-off: ≥730x10e3/L) and higher baseline neuthrophil counts (cut-off: ≥4330x10e3/L) was found on PFS (p for interaction = 0.047) but not on OS (p for interaction = 0.12), with a longer median PFS for those patients with lower neutrophil early Δ (<730x10e3/L) and higher baseline neutrophil counts (≥4330x10e3/L) (HR = 1.76; 95% CI: 1.23-2.52; p = 0.002) (**Figure 6S**). No significant interactions between NLR early Δ (cut-off: ≥0.5) and baseline NLR values (cut-off: ≥3.2) were found in both PFS and OS (p for interaction = 0.36 and 0.89, respectively), suggesting that the association between NLR D, PFS, and OS was similar in patients with baseline NLR below or above the cut-off of 3.2 (**Figure 7S**).

## 4 Discussion

In the era of tyrosine kinease inhibitors (TKIs), ICIs and their combinations for mRCC, baseline clinical, and laboratory characteristics of patients incorporated into the IMDC score (37, 38) still represent the critical factors clinicians consider for the treatment decision making (2, 3, 39). More recently, we proposed implementing the IMDC prognostic stratification by the Meet-URO score, which was demonstrated in large series (34, 40) to be more accurate than IMDC alone by two additional independent prognostic factors (the presence of bone metastases and the NLR). Tumor biomarkers, like the PD-L1 expression or the TMB, have not showed yet a clinical utility, particularly for the ICIs (8, 9), nor dynamic biomarkers, whose variations during treatment might early indicate the tumor sensitivity or resistance, are available. Moreover, early predictors of disease progression could spare patients from ineffective treatments and their related toxicity and could potentially improve patients’ outcomes by allowing an earlier change of treatment line (41).

The early variations of inflammatory indices from peripheral blood are captivating dynamic biomarkers as they have consistently shown their prognostic value in several tumor types and treatment settings in addition to their easy and relatively inexpensive assessment and reproducibility in clinical practice (21, 22, 34). Evidence is accumulating regarding the prognostic value of their early variations, mainly involving the NLR, in advanced non-small-cell lung cancer (23, 25, 29–31), small-cell lung cancer (33), esophageal squamous cell carcinoma (32), and mRCC treated with ICIs (24, 28). However, the mechanisms underlying the dynamic variations of inflammatory indices from peripheral blood during treatments and whether they reflect a change in the immunological status in response to treatment, especially to ICIs, are still unclear.

On these premises, the results of this pre-specified secondary analysis of the Meet-URO 15 study (34), focusing on the quantitative variations of cellular counterparts of the mainly used IR (or the NLR, SII and PLR), provided us with the following four key observations. Firstly, during the initial treatment with nivolumab, there was a consistent neutrophil and relative NLR increase. Secondly, patients with higher platelet and lower lymphocyte baseline counts, and increasing neutrophil counts during the ICI, were more likely to develop disease progression than response. This may also explain why all the baseline IR values but only increasing NLR and SII (i.e., not the PLR) were predictive of PD. Thirdly, survival outcomes (both OS and PFS) were worse for patients with baseline lower lymphocytes and higher platelets (and consequently higher NLR, SII and PLR), and early neutrophil increase over treatment. The latter was particularly relevant in patients with higher baseline neutrophils. Finally, besides baseline NLR and the other known prognostic variables, early rise in neutrophils and NLR resulted as independent prognostic factors on both OS and PFS.

Increased peripheral neutrophils promote tumor development, invasiveness, metastasis, and resistance to treatment (42). The intra-tumoral neutrophil count is also directly related to blood neutrophils (43). Blood lymphocyte counts are associated with the immunological response to malignancy. As a result, the body’s capacity to inhibit cancer cells may be impacted when inflammation results in prolonged lymphocytopenia, including CD4+ and CD8+ T lymphocytes (42, 44). The contribution of lymphocytes from peripheral blood, and their early increase, to the tumor response in mRCC patients treated with immunotherapy was already demonstrated with interleukin-2 treatment (45). Platelets promote an immunosuppressive tumor microenvironment (TME) in addition to tumor-induced aggregation and clotting by secreting angiogenic and mitogenic growth factors and immunosuppressive cytokines and physically shielding tumor cells from cytotoxic lymphocytes and natural killer (NK) cells invading the tumor (46). In addition, they recruit leukocytes to tumor sites and regulate responses of the adaptive immune system (47). NLR may work as a stand-in for tumor inflammation and most likely reflects the suppression of T-cell proliferation by myeloid-derived suppressor cells (MDSC) (48).

The current analysis could not assess the predictive role for immunotherapy of baseline levels or dynamics of peripheral-blood parameters based on neutrophil, lymphocyte, and platelet absolute cell counts, particularly regarding their potential correlation with TME or whether they corresponded to the intratumoral immune response modifications favored by ICIs. For those issues, we should have had a TME correlate and a control arm. Thus, we cannot provide a mechanistic link between the different immune-inflammatory cell populations in the peripheral blood and TME. Moreover, it was not the scope for the current analysis, which focused on the only prognostic value of those blood baseline and dynamic peripheral-blood immune or inflammatory cells and their derived ratios based on their association with survival outcomes of patients with mRCC following immunotherapy. Nonetheless, we believe the findings retain a relevant clinical utility for their exclusive prognostic value while hypothesis-generating for future translational, correlative, or comparative studies. For instance, their routine assessment could represent a helpful tool to predict treatment resistance early. In fact, outside clinical trials, the first radiological disease evaluation is rarely performed earlier than 3 months after the treatment start. Thus, the early increase of neutrophils and NLR, just at the second ICI administration, might prompt the clinician to anticipate the radiological reassessment, thus saving toxicity to patients and the health system and offering the patient a different treatment before clinical worsening would make it not possible, or informing novel prospective adaptive studies with arm allocation based on treatment response (49, 50). Notably, before ICIs and their combinations were used as the first-line treatment, only 42%–57% of mRCC patients were estimated to receive a second-line therapy, and this proportion might have not dramatically increased (51, 52).

We acknowledge as study limitations the retrospective data and analysis (including missing clinical information interplaying with ACC and IR, like comorbidity and steroids, or other concomitant drugs), the possible selection bias (as enrolled patients had to receive at least two nivolumab administrations), the variable timing and clinician-lead disease reassessment (which might have impacted on the definition of disease response), the restriction to variations of ACC as components of the IR (i.e., albumin, lactate dehydrogenase, C-reactive protein, and other inflammatory parameters were not considered), which make more important an external validation of our findings. Another relevant study limitation is the disused treatment setting for immunotherapy. However, the proof-of-principle value of the analysis may be retained. Baseline values and early variations of peripheral blood inflammatory ratios and their cellular components were associated with the clinical outcomes of pretreated patients with metastatic renal cell carcinoma receiving single-agent immunotherapy. It needs confirmation in the front-line setting with immunotherapy-based combinations for which we planned *ad hoc* analyses. Immortal and lead time biases are further analysis limitations related to the variation of blood inflammatory ratios and their dynamic assessment. However, we had a relatively low proportion (7.4% of patients) who did not reach the second treatment cycle, and most patients were treated in the second-line setting. Regarding the immortal time bias, early deaths due to disease progression would be expected in patients with high delta values of blood inflammatory ratios, thus not changing the observed effect direction. Furthermore, the late dynamics of ACC and IRR and their association were not investigated.

Nevertheless, this study is one of the largest reports on the dynamics of inflammatory indices from peripheral blood during treatment with ICIs. It adds biological insights to the prognostic value of IR based on the different baseline and early value variations of their specific cellular components. Moreover, it pointed out the early variation of neutrophils and NLR as new prognostic factors with clinical utility for on-treatment decisions, thus offering a new dynamic non-invasive, routinely available tool, at no additional costs, to help clinicians with early on-treatment decisions concerning patients with mRCC treated with ICIs.

## Data availability statement

The raw data supporting the conclusions of this article will be made available by the authors upon reasonable request.

## Ethics statement

The studies involving human participants were reviewed and approved by Regional ethical committee of Liguria - registration number 068/2019. The patients/participants provided their written informed consent to participate in this study.

## Author contributions

Study concept and design, SR, AS, MSt, GB, GF, and SB; GB and GF contributed equally as senior authors; SR, AS, and MSt contributed equally as first authors. Acquisition and curation of data, all authors; statistical analysis, AS; interpretation of data, SR, AS, MSt, GB, GF, and SB; drafting of the manuscript, SR, AS, MSt, and GB; critical revision of the manuscript for important intellectual content: SR, GB, GF, SB, and DS; supervision, SR, GB, and GF. All authors have read and agree to the published version of the manuscript.

## Funding

This work did not receive any direct funding for conducting the study. SR won the “Giovanni Gardin Award – AIOM Liguria” for this project in September 2019.

## Acknowledgments

SR and GF would like to thank the Italian Ministry of Health (Ricerca Corrente 2018–2021 grants) that financially support their current research focused on identifying prognostic and predictive markers for patients with genitourinary tumors. All authors would like to thank the Italian Network for Research in Urologic-Oncology (Meet-URO).

## Conflict of interest

SR received honoraria as a speaker at scientific events and travel accommodation from Amgen, GSK, BMS, and MSD. GB reports personal fees from AstraZeneca, Janssen-Cilag, Boehringer Ingelheim, Roche, and non-financial support from BMS, AstraZeneca, MedImmune, Pierre Fabre, and IPSEN. GF services advisory boards for Astellas, Janssen, Pfizer, Bayer, MSD, and Merck and received travel accommodation from Astellas, Janssen, and Bayer. SB received honoraria as speaker at scientific events and advisory role by BMS, Pfizer, MSD, Ipsen, Roche, Eli Lilly, AstraZeneca, Pierre-Fabre, and Novartis. DS received honoraria for the advisory board from Amgen, Jansen, MSD, BMS, Bayer, Astra Zeneca, Ipsen, Novartis, and Merck. UG serves as advisory/board member of Astellas, Bayer, BMS, IPSEN, Janssen, Merck, Pfizer, and Sanofi, and received research grant/funding to the institution from AstraZeneca, Roche, Sanofi and travel/accommodations/expenses from BMS, IPSEN, Janssen, and Pfizer. PZ services advisory boards/consulting for Pfizer, BMS, MSD, IPSEN, Novartis, Roche, Amgen, AstraZeneca, Sanofi, Janssen, and Astellas. GPro received a personal fee for consulting or advisory role AstraZeneca, Bayer, BMS, Eisai, Janssen, Ipsen, Merck, MSD, Novartis, and Pfizer and a research grant from Astellas, Ipsen, Novartis. MSo received honoraria as consultant or advisory role from Janssen; grants for participation at scientific events from Ipsen, Janssen, Bristol Myers Squibb, Pfizer, Astellas Pharma, Sanofi, Roche, and Novartis; and research funding from Roche, Merck, Janssen. ACo receives speaker fees/grant consultancies from Astrazeneca, BMS, MSD, Roche, Novartis, and Astellas. FMo received grants from MSD and Pfizer. GR received honoraria for advisory boards or invited speaker fees from BMS, Astellas, Bayer, Ipsen, Novartis, Roche, and AstraZeneca.

The remaining authors declare that the research was conducted in the absence of any commercial or financial relationships that could be construed as a potential conflict of interest.

## Publisher’s note

All claims expressed in this article are solely those of the authors and do not necessarily represent those of their affiliated organizations, or those of the publisher, the editors and the reviewers. Any product that may be evaluated in this article, or claim that may be made by its manufacturer, is not guaranteed or endorsed by the publisher.
