# Supplementary material for: The prognostic value of baseline and early variations of peripheral blood inflammatory ratios and their cellular components in patients with metastatic renal cell carcinoma treated with nivolumab: The Δ-Meet-URO analysis
Source: Front Oncol. 2022 Sep 23;12:955501. doi: 10.3389/fonc.2022.955501 (PMC9541611; doi:10.3389/fonc.2022.955501)
Supplement: Supplementary file 1 [file DataSheet_1.docx]

Supplementary Material

# Supplementary Tables

**Table 1S. Multivariable analysis on PFS of absolute cell counts and immune-inflammatory indices baseline and early** Δ**, and baseline clinical parameters.**

| **Inflammatory indices** | **ROC-based cut-off values** | **Multivariable Cox regression for PFS** | | |
| --- | --- | --- | --- | --- |
|  |  | **NLR**  **(HR; 95% CI;**  ***p* value)** | **SII**  **(HR; 95% CI;**  ***p* value)** | **Neutrophils**  **(HR; 95% CI; *p* value)** |
| Baseline NLR | ≥ 3.2 | 1.54; 1.21-1.96; **p<0.001** |  |  |
|  | < 3.2 | 1.00 (ref) |  |  |
| Early Δ NLR | ≥ 0.5 | 1.51; 1.20-1.91; **p=0.001** |  |  |
|  | < 0.5 | 1.00 (ref) |  |  |
|  |  | *p* value for interaction  baseline NLR and ΔNLR = 0.28 |  |  |
| Baseline SII | ≥ 720 |  | 1.29; 1.01-1.63; **p=0.038** |  |
|  | < 720 |  | 1.00 (ref) |  |
| Early Δ SII | ≥ 218 |  | 1.24; 0.98-1.56; p=0.070 |  |
|  | < 218 |  | 1.00 (ref) |  |
|  |  |  | *p* value for interaction  baseline SII and ΔSII = 0.87 |  |
| Baseline Neutrophil | ≥ 4330 x10^3/μL |  |  | 1.22; 0.96-1.55; p=0.10 |
|  | < 4330 x10^3/μL |  |  | 1.00 (ref) |
| Early Δ Neutrophil | ≥ 730 x10^3/μL |  |  | 1.32; 1.04-1.68; **p=0.023** |
|  | <730 x10^3/μL |  |  | 1.00 (ref) |
|  |  |  |  | *p* value for interaction  baseline Neutrophils and ΔNeutrophils = 0.024 |
| **Clinical parameter** |  |  |  |  |
| IMDC score | Favorable | 1.00 (ref) | 1.00 (ref) | 1.00 (ref) |
|  | Intermediate | 1.74; 1.28-2.37; **p<0.001** | 1.70; 1.25-2.31; **p=0.001** | 1.71; 1.26-2.33; **p=0.001** |
|  | Poor | 2.58; 1.68-3.97; **p<0.001** | 2.52; 1.63-3.91; **p<0.001** | 2.67; 1.74-4.11; **p<0.001** |
| Nephrectomy | Yes | 0.80; 0.56-1.15; p=0.23 | 0.73; 0.51-1.04; p=0.078 | 0.71; 0.50-1.02; p=0.063 |
|  | No | 1.00 (ref) | 1.00 (ref) | 1.00 (ref) |
| Bone | Yes | 1.40; 1.11-1.78; **p=0.005** | 1.33; 1.05-1.69; **p=0.017** | 1.38; 1.09-1.75; **p=0.008** |
|  | No | 1.00 (ref) | 1.00 (ref) | 1.00 (ref) |

*CI* confidence interval, *early Δ* value variations between 2^nd^ and 1^st^ therapy infusion, *HR* hazard ratio, *IMDC* International Metastatic RCC Database Consortium Risk Score for RCC, *NLR* neutrophils-to-lymphocytes ratio, *PFS* progression-free survival, *RCC* renal cell carcinoma, *ROC* receiving operating curve, *SII* systemic immune-inflammatory index. In bold, significant p values.

# Supplementary Figures

**Figure 1S. CONSORT flow diagram**


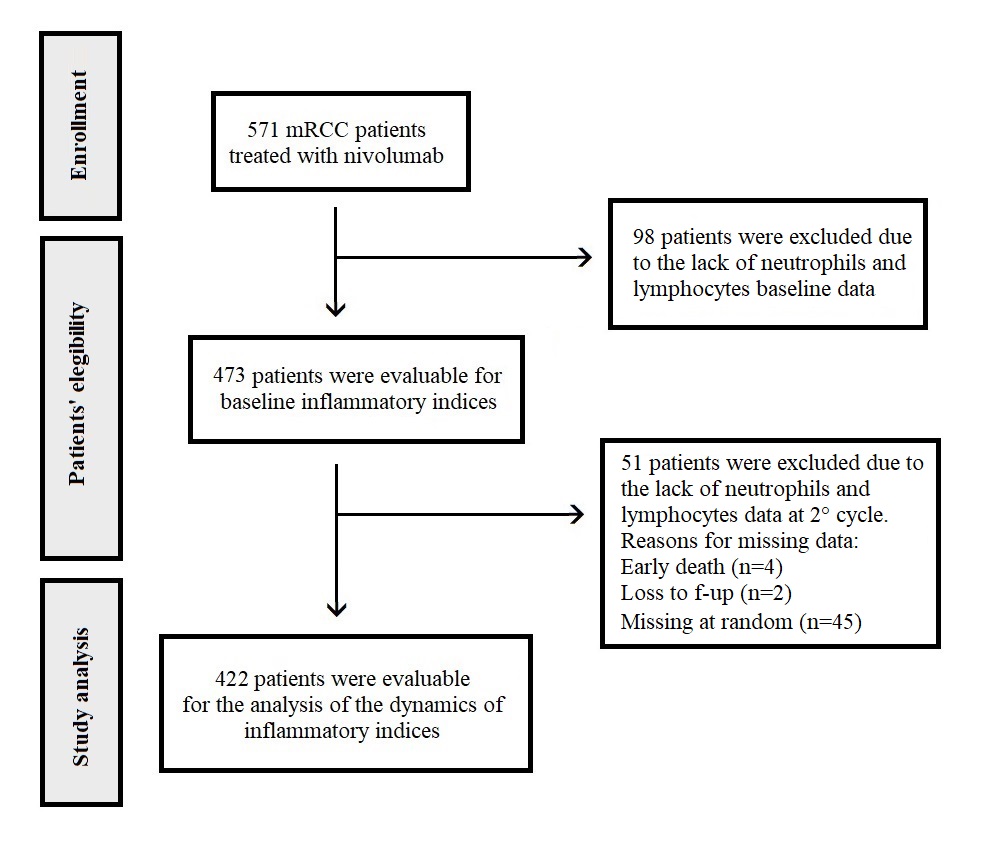


**Figure 2S. The univariable analyses of baseline NLR (A), SII (B), PLR (C) and neutrophils (D), lymphocytes (E) and platelets (F) on progression-free survival.**


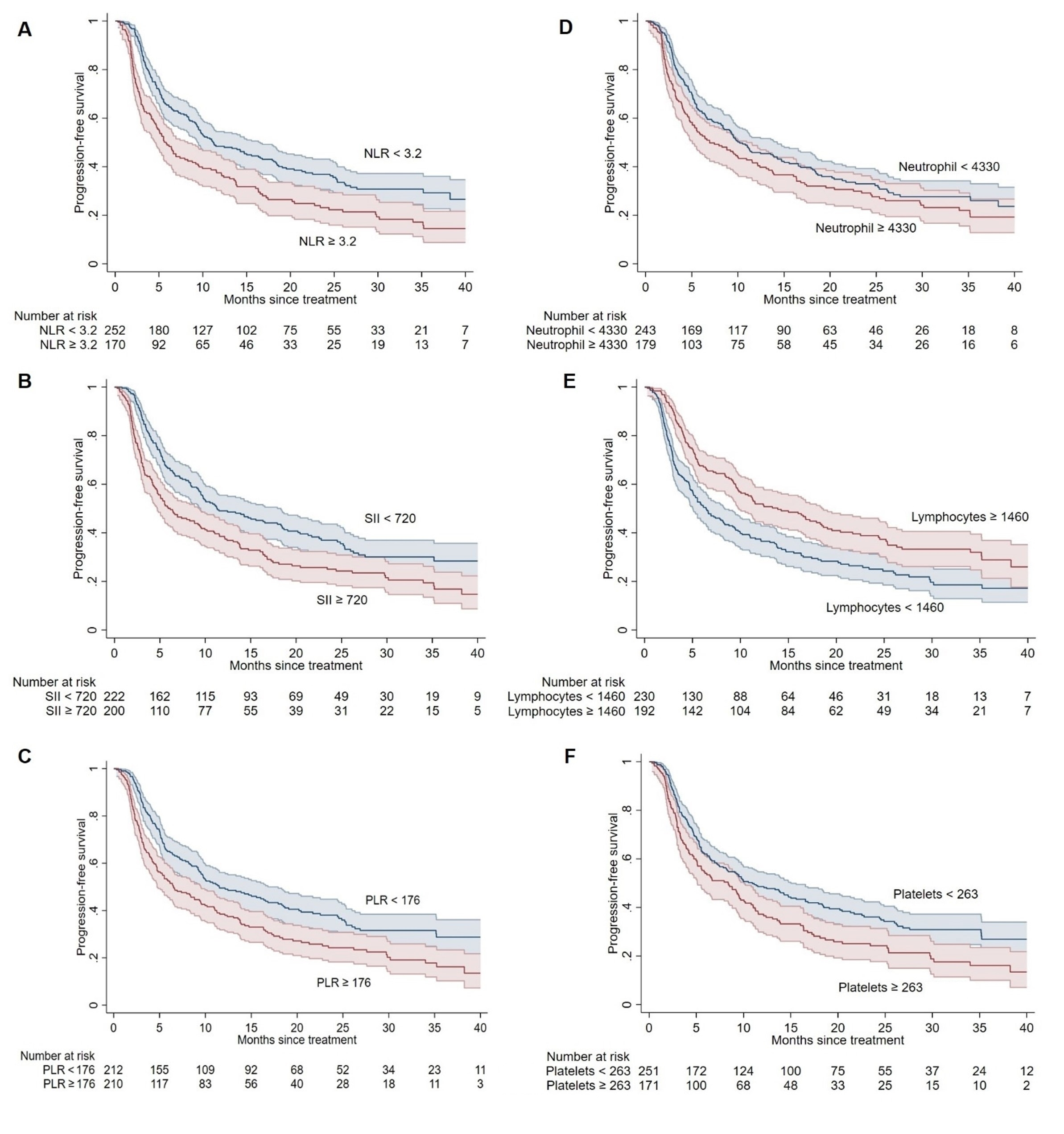


**Figure 3S. The univariable analyses of baseline SII (A), PLR (B), lymphocytes (C) and platelets (D) on overall survival.**


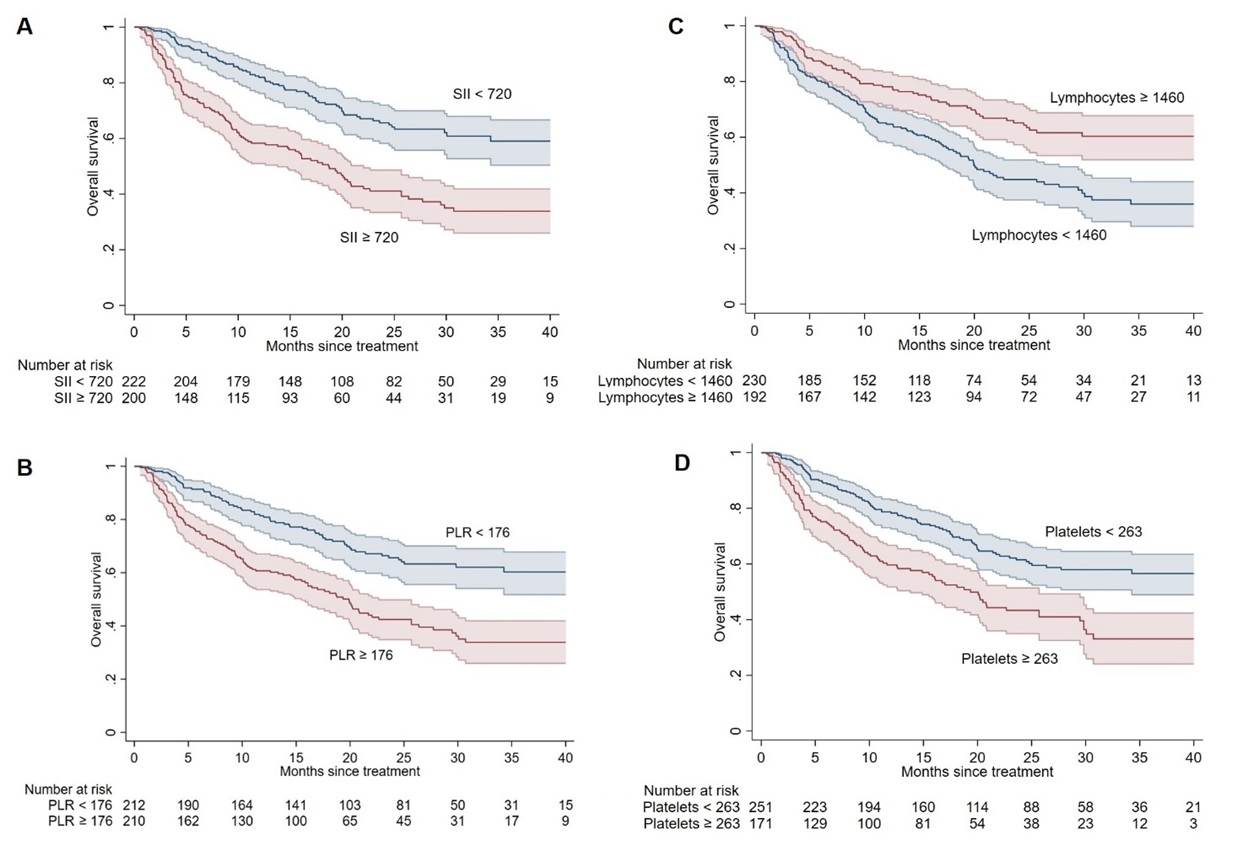


**Figure 4S. The univariable analyses of early D of SII (A), PLR (B), lymphocytes (C) and platelets (D) on overall survival.**


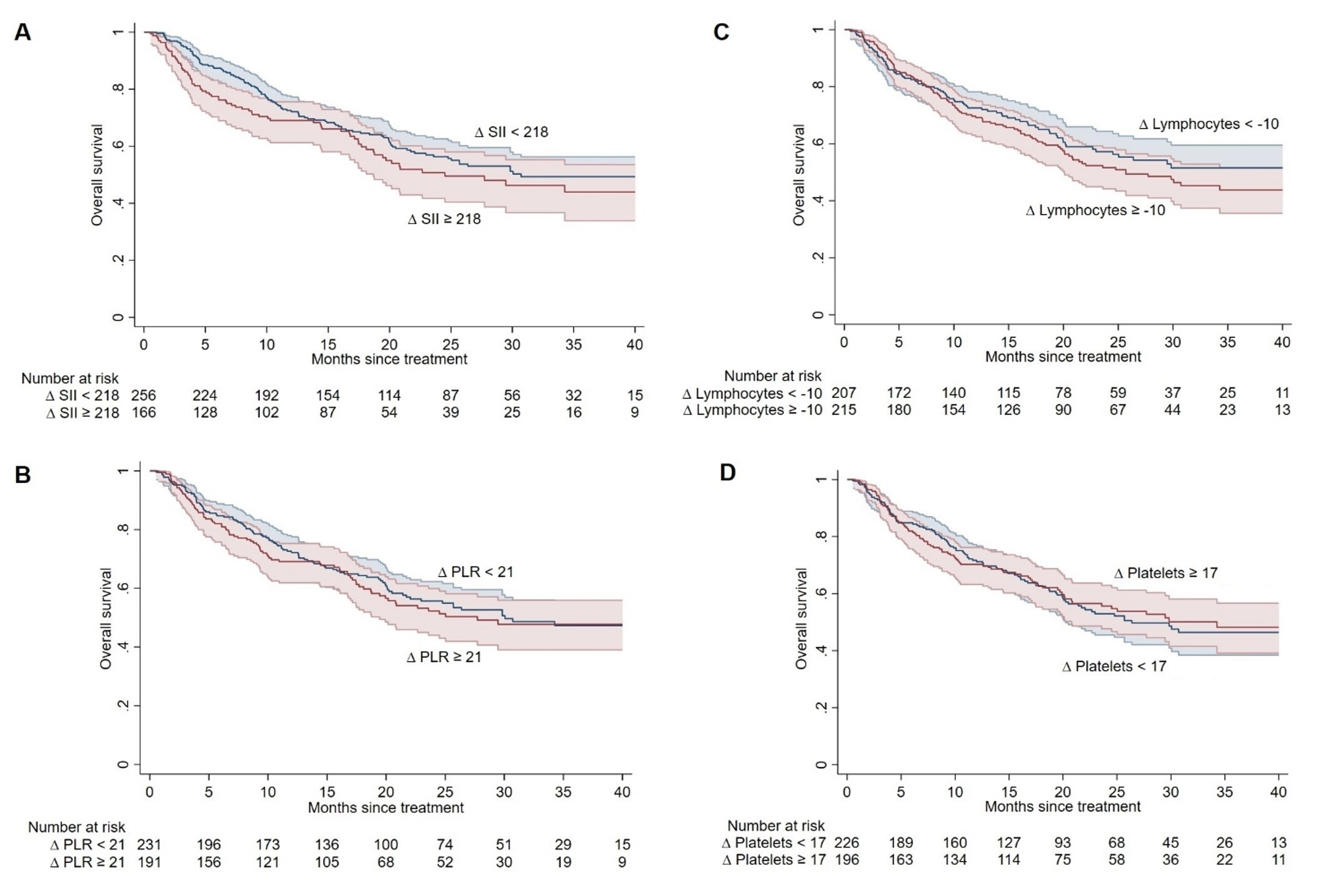


**Figure 5S. The univariable analyses of early D of NLR (A), SII (B), PLR (C), neutrophils (D), lymphocytes (E) and platelets (F) on progression-free survival.**


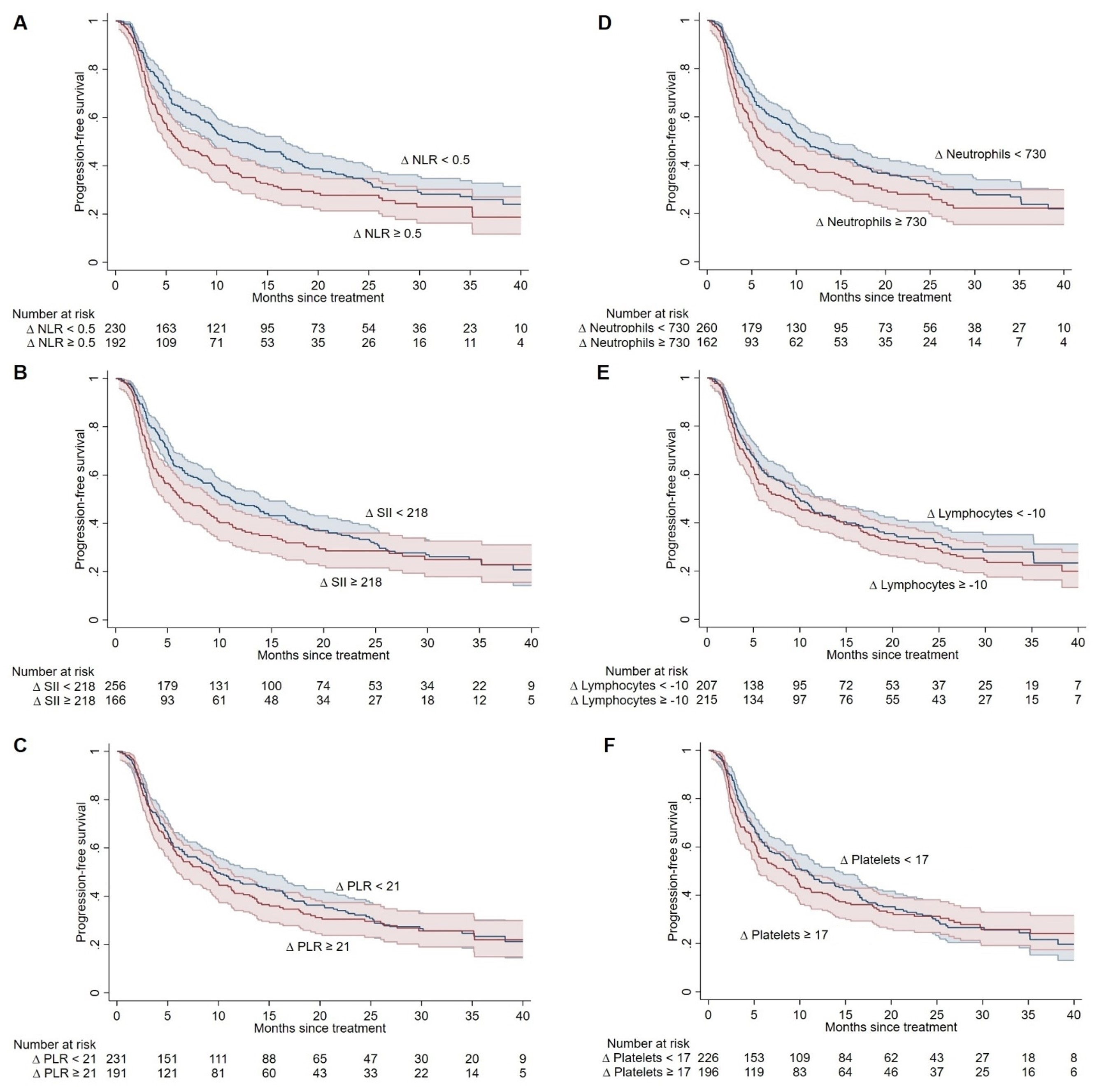


**Figure 6S. The univariable analyses of early Δ of neutrophils according to basal neutrophils values on progression-free survival (A,C) and overall survival (B,D).**

**
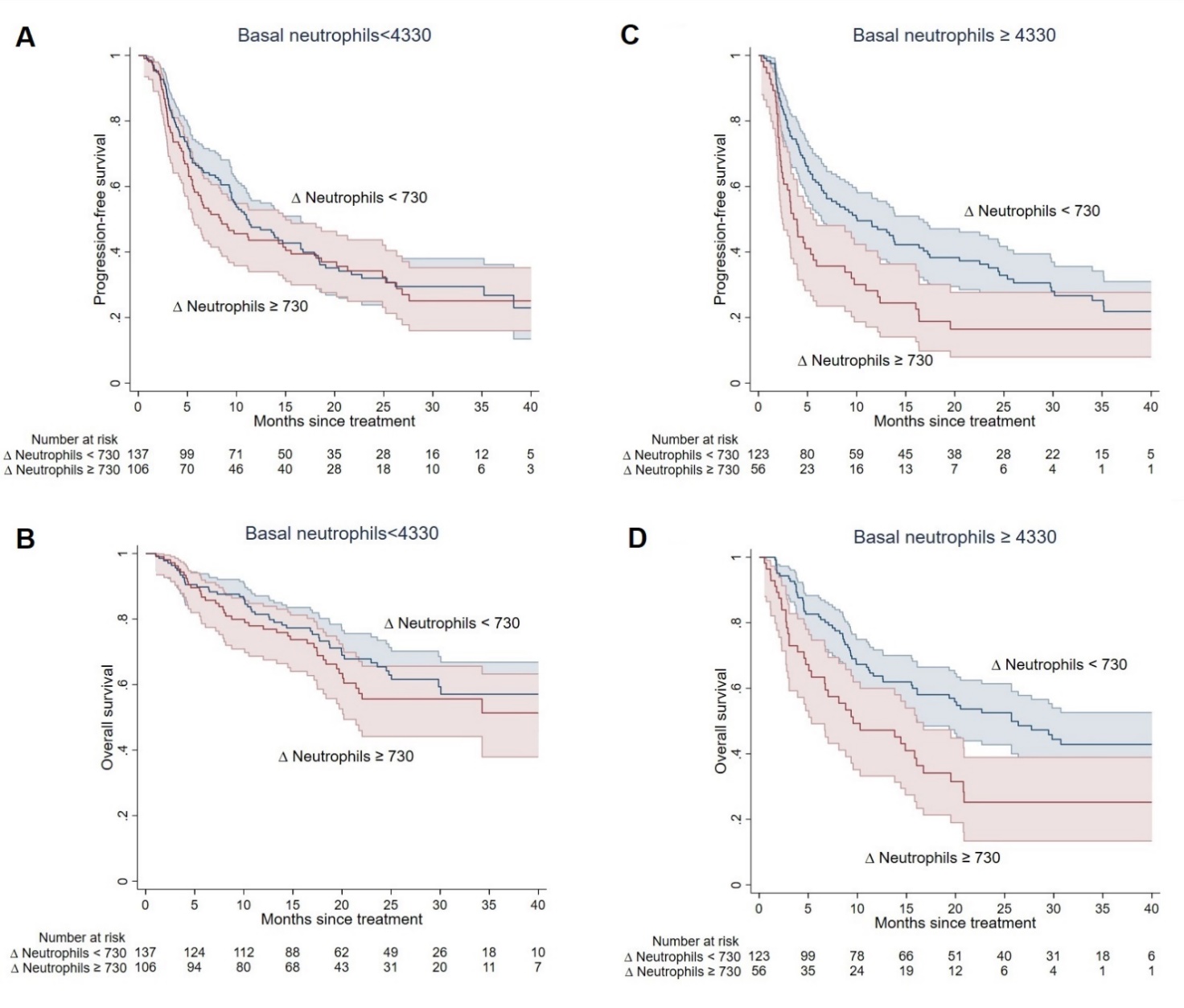
**

**Figure 7S. The univariable analyses of early Δ of NLR according to basal neutrophils values on progression-free survival (A,C) and overall survival (B,D).**

**
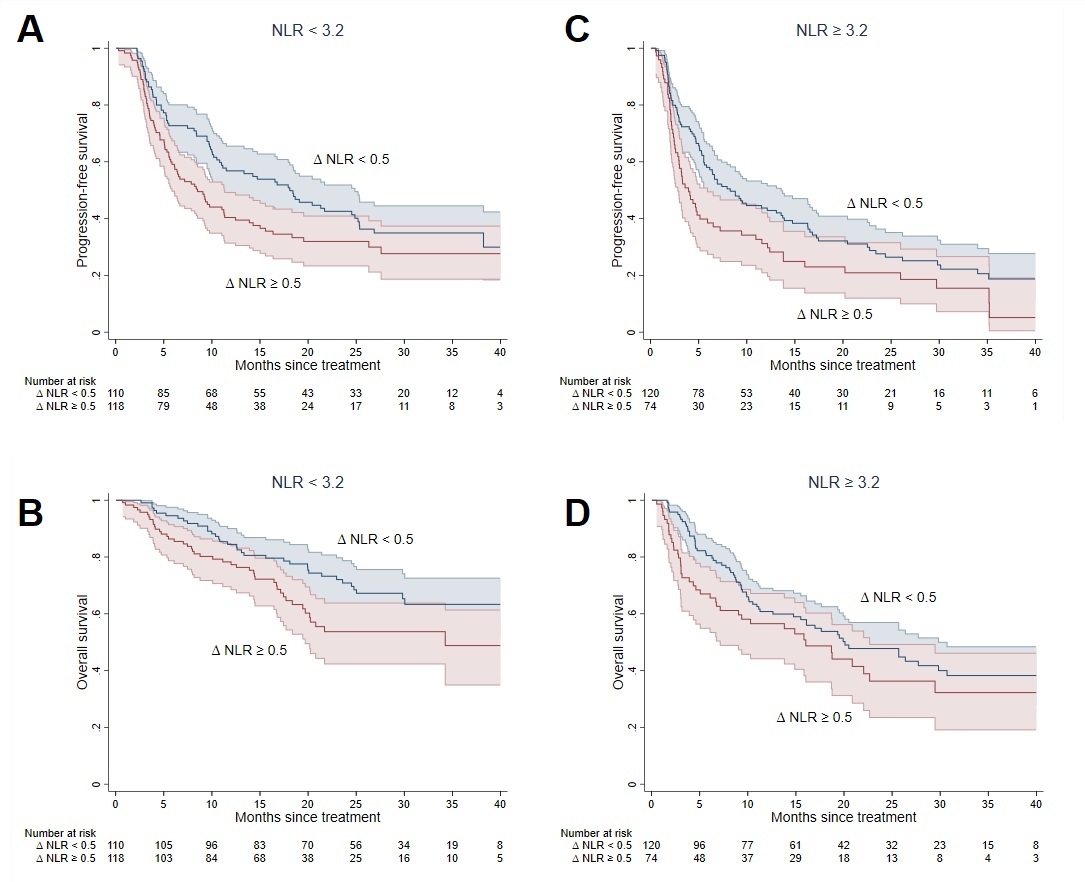
**
